# Setting targets for human resources for eye health in sub-Saharan Africa: what evidence should be used?

**DOI:** 10.1186/s12960-016-0107-x

**Published:** 2016-03-16

**Authors:** Paul Courtright, Wanjiku Mathenge, Amir Bedri Kello, Colin Cook, Khumbo Kalua, Susan Lewallen

**Affiliations:** Kilimanjaro Centre for Community Ophthalmology International c/o Division of Ophthalmology, University of Cape Town, Cape Town, South Africa; Rwanda International Institute of Ophthalmology, Kigali, Rwanda; Dr. Agarwal’s Eye Hospital, Kigali, Rwanda; Light for the World, Addis Ababa, Ethiopia; Division of Ophthalmology, University of Cape Town, Cape Town, South Africa; Blantyre Institute of Community Ophthalmology, Blantyre, Malawi

**Keywords:** Ophthalmologist, Cataract surgeon, Africa, Primary eye care

## Abstract

With a global target set at reducing vision loss by 25% by the year 2019, sub-Saharan Africa with an estimated 4.8 million blind persons will require human resources for eye health (HReH) that need to be available, appropriately skilled, supported, and productive. Targets for HReH are useful for planning, monitoring, and resource mobilization, but they need to be updated and informed by evidence of effectiveness and efficiency. Supporting evidence should take into consideration (1) ever-changing disease-specific issues including the epidemiology, the complexity of diagnosis and treatment, and the technology needed for diagnosis and treatment of each condition; (2) the changing demands for vision-related services of an increasingly urbanized population; and (3) interconnected health system issues that affect productivity and quality. The existing targets for HReH and some of the existing strategies such as task shifting of cataract surgery and trichiasis surgery, as well as the scope of eye care interventions for primary eye care workers, will need to be re-evaluated and re-defined against such evidence or supported by new evidence.

## Background

Vision loss affects approximately 223 million people globally, 32 million of whom are blind [1]; there are an estimated 4.8 million blind in sub-Saharan Africa (SSA) [2]. The World Health Organization (WHO) and the consortium of non-governmental organizations and academic institutions that make up the International Agency for the Prevention of Blindness (IAPB) developed the VISION 2020: Right to Sight initiative in 1999 with the expressed goal of eliminating avoidable blindness by the year 2020 [3]. More recently, WHO has developed the Global Action Plan (GAP) with the goal of reducing vision loss by 25% by the year 2019 [4]. There has been considerable progress in reducing vision loss in SSA [2] as well as tackling specific conditions such as vitamin A deficiency and measles-related blindness, once estimated to be the leading cause of blindness in children [5]. The prevalence of trachoma, the leading cause of infectious blindness, has also dropped considerably in the last 15 years [6]. That said, as the numbers of people estimated blind (defined as presenting vision in the best eye of less than 3/60, the equivalent of not being able to detect the largest E on an eye chart at 3 m) still hover around 32.4 million [1], vision loss and blindness remain significant challenges globally and in SSA.

As the VISION 2020 initiative and the GAP both indicate, the required human resources for eye health (HReH) need to be available, appropriately skilled, supported, and productive. Early efforts sought to establish benchmarks for the number of specific cadres of HReH, particularly for cataract surgery as cataract was, and remains, the leading cause of blindness globally [1]. Targets for SSA were generally based upon the understanding of the epidemiology of blindness and prevalence of cataract at the time. Establishing HReH targets is attractive; they can be used as a guide by national ministries of health to measure current HReH status and to plan for the future. Assessment of current HReH against targets also lends itself to effective fund raising by non-governmental organizations to support training initiatives. Finally, HReH targets provide large consortiums, like IAPB, with a clear goal and indicators to measure progress towards the goal. However, it is important to remember that the targets suggested in 1999 were based on “expert opinion” [3, 7, 8] and there is need to revisit them as new evidence becomes available. In this paper, we do not attempt to set new targets but rather to provide a review of some of the new evidence from SSA that need to be considered since the initiation of VISION 2020 almost 20 year ago.

The growing body of evidence on HReH in SSA also can be used to inform HReH policies. This is critical since resources, both financial and human, remain limited and policies should help ensure that strategies that are proven to work are adopted and that strategies that are shown not to work are discarded. Adoption of HReH policies without consideration of the evidence of effectiveness and efficiency can lead to a frustrated workforce, a frustrated population (not getting the service desired), and can embed practices that are regressive rather than progressive in nature. Investment in HReH, whether by government agencies or by consortia like IAPB, has long-term implications. Thus, providing the information and evidence needed to inform policy-making should be a priority for all concerned.

A variety of abnormalities can affect the eye including vascular, inflammatory, traumatic, metabolic, infectious, nutritional, degenerative, congenital, or combinations of these. In addition, minor shape and size discrepancies in otherwise healthy eyes result in refractive errors. Any of these etiologic factors may lead to impaired vision. The eye is a complex organ, and its structures are affected differently by the various etiological factors. Accurate diagnosis depends largely on examination and visualization of the affected structures and much less on history or laboratory findings. However, the eye is small and sophisticated and usually expensive instruments are needed to examine it properly. Considerable practice is needed to master the examination techniques. The implications for human resource needs are considered below for the major causes of blindness and visual impairment.

In SSA, there are several cadres who provide eye care. These are often divided into primary-, middle-, and tertiary-level personnel, although the precise skills, competencies, and training of the primary and midlevel workers are not consistent across the continent. The following is a summary of the cadres, noting the areas where inconsistencies are common.

Ophthalmologists are medical doctors who, after general training in medicine, have specialized in eye diseases and their management. They perform surgery and provide medical treatment for diseases that affect the eye. In Francophone countries, only those with extra training provide surgery while all Anglophone ophthalmology training programmes in SSA include surgery.

Midlevel personnel mostly comprise clinical officers and nurses who have specialized in ophthalmology after working as general health care providers for a few years. These cadres are usually, although not always, recognized as specialist workers by the ministry of health (MoH). Their ophthalmology training varies from 1 to 3 years, and they are usually “dedicated” eye workers, i.e. they spend all of their time providing eye care. Nomenclature varies but the terms most commonly used include ophthalmic clinical officers or ophthalmic nurses. Some of these are trained to do cataract surgery (non-physician cataract surgeons (NPCS)); however, surgery by non-physicians is expressly forbidden in some countries (e.g. Nigeria, South Africa, Rwanda) and NPCS are not used in these. [9]. Optometrists are a cadre without background in health care who are trained to provide refractive services and glasses. Recognition of this cadre and their training in SSA have been highly variable. Recently, with support from non-governmental organizations, new optometry-training programmes have been established in several countries in SSA. These also provide shorter training modules varying from 3 to 12 months to become a “refractionist”. Ophthalmic clinical officers and nurses are also trained in refraction and have provided the bulk of these services in SSA. The need for specialized refraction personnel is discussed more below in the section on epidemiology of refractive error.

Primary eye care personnel in SSA are general health workers at the primary care facilities. This cadre, in theory, is supposed to provide treatment or referral for anyone who comes from the community with eye complaints. They are expected to serve as the frontline in providing eye care, in addition to their other duties such as providing immunization, malaria, maternal child health, HIV/AIDS care, and other services. Many have a short module (a few days) on “eye care” in their basic training. In addition, a number of NGOs run courses (varying from a few days to a few weeks) in “primary eye care” for this cadre. MoH in most countries do not recognize any special cadre of “primary eye care workers”; the push has been to “integrate” primary eye care into general health care services. The role of community health workers has generally remained in the important sphere of eye health education and promotion.

## Evidence needed for HReH policies and target setting

The type of information and evidence that is needed to inform HReH policy and set targets can be grouped, broadly, in three categories: (1) disease-specific issues including the epidemiology, the complexity of diagnosis and treatment, and the technology needed for diagnosis and treatment of each condition, (2) population needs and demands for vision-related services, and (3) health system issues, primarily related to the HReH to provide these services. Each of these three categories is dynamic, changing with socio-economic and other development parameters; this means that recommendations today may not be appropriate in SSA in 10 years’ time, just as recommendations at the initiation of VISION 2020 need re-examination today. We will consider each of these three “categories” separately reviewing current evidence relevant for HReH planning and policies.

### Disease-specific issues

In the past, there was much focus on infectious eye diseases in SSA, such as trachoma, onchocerciasis, and vitamin A deficiency; these are conditions that can and should be prevented by community-based public health measures. Prevention of these does not require much, if any, specialized knowledge of ophthalmology. Successful public health programmes have resulted in a decrease in these conditions; now they exist only in pockets and contribute very little to the overall burden of vision loss in SSA [2, 5]. The most common causes of vision loss are cataract, refractive error, and posterior segment diseases (comprising chiefly glaucoma, diabetic retinopathy, and age-related macular degeneration). While there is no primary prevention for these conditions, the first two and some of the posterior segment diseases can be treated with a high degree of success. In considering HReH in Africa, it is critical to understand something about the epidemiology of these specific conditions as well as the technical requirements to diagnose and treat them.

#### Cataract

For many decades, SSA suffered from a lack of sound population-based data on cataract, forcing extrapolations across large sections of the continent from only a few surveys—and these not necessarily carried out in “representative areas”. Newer survey methodology, however, has allowed a much better understanding of the epidemiology not only of blindness and visual impairment in SSA but of cataract epidemiology in particular. Analysis of these new survey data indicates that the epidemiology of cataract varies substantially across SSA; importantly, the age-adjusted incidence is two to four times higher in Sahel populations (roughly corresponding to the Nilo-Saharan and Afro-Asiatic language groups) than in Bantu language group populations [10]. This may be related to genetic variations or to environmental or cultural factors; whatever the underlying explanation, the variation clearly has implications for HReH needs, which should be expected to vary across Africa.

Treatment of cataract is by surgery, removing the clouded lens and inserting an artificial intraocular lens (IOL). The techniques to do this safely and effectively and to restore vision completely have improved by leaps and bounds in the last two decades and continue to evolve. This means that well-trained and equipped surgeons can offer treatment to people with cataract vision loss far earlier, confident that they will restore normal vision to those with even a small amount of clouding in the lens. However, making sure that vision loss in a given patient is truly due to a “small amount of clouding” and not some other condition requires sophisticated equipment and diagnostic skills that only a health worker specialized in eyes would have. This is very different from the past, when surgery was restricted to eyes with advanced “white cataract”; starting from a condition of such extreme loss of vision, patients were likely to get *some* improvement post operatively, even if the surgery was not perfectly executed.

Regarding the personnel who perform cataract surgery, studies have demonstrated that there is a vast variation in their productivity, with some providing fewer than 100 cataract surgeries per year while others provide thousands [11–14]. In the most recent study [13], the mean number of surgeries per surgeon per year was 188 but the median was only 76, the range being from 0 to 1 700. The factors that allow high productivity have been studied and enumerated but are beyond the scope of this paper to describe in detail. One of the factors at the hospital level is enough trained support personnel; this means midlevel personnel in the operating theatre to streamline the surgery, midlevel personnel in the clinics to ensure a steady supply of cataract patients to the surgeon, and non-medical management personnel to keep the eye service running efficiently [12–14]. Outside the hospital, a factor that influences surgeons’ productivity is having a system that reaches into the communities to identify and recruit patients and bring them into contact with the surgeon. Clearly, such factors should be critical considerations in setting “targets” for HReH. In an efficiently operating system, it is easily possible for a surgeon to perform 10 operations per day; assuming that two surgical days per week are allocated for cataract surgery and a surgeon works 40 weeks in a year, the target of 800 operations per surgeon per year is not unreasonable. The number of procedures is not the only measure of productivity, but it is likely the most important measure.

#### Refractive error

Globally, refractive error is the leading cause of visual impairment (other than blindness) [15]. There is huge variation in different parts of the world in the prevalence of myopia (inability to focus on distant objects); while it is very common in school-age East Asian populations, significant myopia remains a relatively rare condition in SSA [16] and blindness from refractive error in adults is very rare [17]. There are no reliable data on possible variations in refractive error prevalence across SSA, and this question deserves study as it could have implications for HReH needs at a subregional level. A recent global study of the economic impact of correcting refractive error in adults concluded that, due to the low prevalence of refractive errors in Africa, large investment in programmes to address refractive errors in SSA is not indicated at the present time [18]. The GAP [4] has an indicator concerning the number of optometrists/refractionists per million per member state, but it seems unreasonable to suggest that the number of refractionists/optometrists needed in SSA would be the same as that needed elsewhere in the world.

Presbyopia, the loss of ability to focus on near objects that occurs with aging, is considered a universal problem, and there is no compelling evidence that its epidemiology varies significantly in SSA compared to the rest of the world. It should not require specialized eye care personnel to manage; however, its management could provide an important opportunity to diagnose potentially sight-threatening conditions such as glaucoma and diabetic retinopathy.

Diagnosis and management of refractive errors has not changed significantly in the past 20 years in SSA. It is well suited to be carried out by midlevel personnel. In SSA, the training of midlevel eye workers (nurses, clinical officers) usually includes refractive error, and where this cadre exists, it is not clear that a special cadre of refractionists is required.

#### Posterior segment diseases

Diseases that affect the back of the eye (posterior segment eye diseases) are emerging as important threats to people’s vision in SSA. These diseases, which include diabetic retinopathy, glaucoma, and age-related macular degeneration become more prevalent with age, and the demographic transition in SSA as well as the growing number of diabetics means there is an increase in those affected [19, 20]. Unlike cataract, these are chronic diseases which often result in irreversible vision loss if left undetected. They require a high degree of skill and sophisticated equipment to diagnose and manage.

In surveys, posterior segment diseases are often grouped together, and in SSA, they account for 13–37% of blindness and visual impairment [21]. The prevalence of glaucoma varies throughout Africa with southern Africa generally reporting the highest age-specific prevalence figures [2, 22, 23]; the prevalence of diabetic retinopathy depends, of course, on the underlying prevalence of diabetes. Glaucoma was not included as a priority at the inception of the VISION 2020 initiative because it was considered too difficult to manage as a public health issue, i.e. there is no practical method for population screening, no simple definitions for what constitutes disease needing treatment, and variably successful treatment options. None of those considerations has really changed, but the growing realization of the magnitude of the problem is spurring suggestions that glaucoma must be addressed. There are few studies of models utilizing the primary and secondary levels to manage glaucoma although there are reports of strategies that have *not* worked [24]. Diagnosis and management of diabetic retinopathy calls for eye care services that are well integrated into the general health system where diabetics are treated. Again, specific roles for various cadres within the HReH are not defined although various models are being tried [25]. An interdisciplinary approach involving primary health care workers, physicians, and eye care workers is probably needed to prevent vision loss from these chronic diseases and to improve quality of life for those with irreversible vision loss; however, successful integrated models with clear roles for the various personnel have not been widely demonstrated in SSA. What we do know is that human resources with specialized clinical knowledge of eye conditions and equipment and resources are needed to provide services. Ophthalmic technicians are needed in performing optical coherence tomography (OCT), visual fields, and angiographies while ophthalmologists are needed for arriving at the proper diagnosis and management decisions including surgical interventions such as trabeculectomies, anti-VEGF injections, and vitrectomies.

#### Vitamin A deficiency/measles, onchocerciasis, and trachoma

Conditions such as vitamin A deficiency and measles-related corneal problems, trachoma, and onchocerciasis were once considered synonymous with blindness in SSA. These were the conditions that originally inspired the concept of “primary eye care” [26] because they lend themselves to action by general health workers at the community level. Successful broad-based measles immunization and vitamin A supplementation efforts throughout Africa and focal large-scale initiatives against trachoma and onchocerciasis mean that these conditions are rapidly decreasing as causes of vision loss [5, 6]. Trachoma control programmes (now usually under neglected tropical diseases in government health systems) require interaction with eye care service providers in order to ensure that trachomatous trichiasis (inturned lashes) are operated, but otherwise, control efforts for these conditions do not require significant interaction with eye care programmes and providers. Nonetheless, there are lessons to be learned about providing eye care from the experiences described below in “task shifting” eye care to general health workers for trichiasis surgery.

With reductions in blindness due to vitamin A deficiency and measles, surgical conditions such as congenital and developmental cataract have become the focus of child eye care programmes in SSA [27]. These require a more sophisticated service delivery model and Child Eye Health Tertiary Facilities are the focus of intervention in many settings [28].

### Patient demand for eye care services

Adults usually request eye care services because of vision loss (distance vision or near vision) or because they have an acute condition (e.g. red, painful eye, trauma) or because efforts at patient education have been successful (e.g. screening of diabetic patients or first-degree relatives of patients with glaucoma).

Visual needs vary tremendously depending on occupation, education, and whether one watches TV, drives, or uses a mobile phone. These factors are changing and leading to increased demand for cataract surgery earlier, before blindness or severe visual impairment occurs [29]. Naturally, demand first increases in urban areas, but when well-trained surgeons operate earlier, it is expected that demand will also accelerate in rural areas. In fact, counter intuitively, those with the most advanced cataract are often least likely to desire surgery [30]. In settings where only the cataract blind are offered surgery, the large number of people desiring intervention before this stage becomes frustrated and often distrustful of the service. Those that have the financial resources may travel to large cities or even outside of the country to receive the service.

At the same time, strategies to generate demand and educate the public about treatment options are essential for effective eye care services. People have to be educated to know that vision loss is not inevitable with aging and that there are effective treatments. Diabetic patients need counselling to seek annual ophthalmologic screening [31], and first-degree relatives of glaucoma patients need to be encouraged to seek glaucoma assessment [32]. Parents need to know that a “white pupil” in a child is never normal and that a specialized eye health worker needs to examine the child urgently.

There is considerable interaction between the epidemiology, diagnosis, and management of eye conditions and the patient demand for eye services. As diagnosis and management improves, patient demand increases. Where the incidence of vision-impairing cataract is higher at younger ages, there will be higher demand for early cataract surgery; generally, the working-age population seeks intervention prior to the onset of severe visual disability. This group is particularly demanding of good outcomes from cataract surgery.

### Health system issues

In the public sector in SSA, eye care is generally situated as a separate unit within general hospitals; most services provided are curative since there are few conditions that can actually be prevented. As noted by previous authors [33], the six subsystems (“building blocks”) of the health system architecture are interdependent; service provision is strongly linked to human resources, equipment and supplies, financing, information flow, and governance at the implementation level. Weaknesses in one impact the others. The foundation for most discussion of health systems for eye health is on the human resources available, often still focused on their number rather than productivity or quality of services [11, 34–36]. At the inception of the VISION 2020 initiative, an attempt was made to estimate how many ophthalmologists and other eye care workers were needed in SSA. There was a range suggested, from one to four ophthalmologists per million [3, 36]. Considering that these were the first ever estimates made, it is understandable that they would range so widely. However, such a wide range (400%) is not really helpful as countries get closer to reaching targets. The more recent GAP suggests that four ophthalmologists per million are needed [4], but exactly how this figure was justified is not clear. Palmer et al. [11] documented that SSA has about 2.9 surgeons per million population (about three fourths of the GAP target) while the productivity (cataract surgeries per surgeon per year) was quite low, only about 178 per surgeon per year.

In response to the perceived shortfall of ophthalmologists in many SSA countries compared to the suggested target, a task shifting strategy, whereby non-physician cataract surgeons (NPCS) are trained, has been embraced by countries such as Tanzania, Kenya, Malawi, and the Gambia. The intent of NPCS training was as a stop-gap measure while countries established medical schools, trained doctors, and then trained ophthalmologists. NPCS training usually occurs in formal 1–2-year programmes supported by NGOs and MoH. The expectation was that NPCS would be placed in rural locations, where there were no ophthalmologists to provide cataract surgery. In spite of some calls to increase this training [37], there is little evidence to indicate that simply scaling up existing training schemes is a good solution. The productivity of NPCS varies hugely [12–14]. Other factors such as retention, quality of care, and service in underserved areas must be considered in evaluating the success of NPCS. Research on NPCS in eastern Africa indicates that the number of cataract surgeries per year per surgeon is relatively low, 243 when measured in 2007 [12] and 188 when measured in 2013 [13]. The one study, in Ethiopia, that included both NPCS and ophthalmologists found that the productivity of NPCS (surgeries/surgeon/year) was 280 compared to 682 for ophthalmologists in the same area [14]. There are a number of reasons for the low productivity: NPCS are generally trained to do surgery on “cataract blindness” rather than on people before blindness, and NPCS have limited capacity to negotiate the necessary support (manpower, supplies, financing) required to provide the surgical services compared to physicians. In some countries, they have been placed in settings in which the catchment population is too small to generate adequate numbers of patients [13]. Concerns with the outcome of surgery have been expressed, but the lack of regular monitoring of outcomes performed by either NPCS or ophthalmologists means that no conclusions can be drawn. While NPCS are more likely to be in rural areas compared to ophthalmologists [11], 65% of NPCS in SSA work in a setting with an ophthalmologist, an example of task sharing [9].

Task shifting has also been tried to address a perceived shortage of eye workers to operate on eyelids for trachomatous trichiasis. In this case, the surgery was shifted from midlevel eye workers to general health workers, and many hundreds of this latter cadre were trained. This initiative was undertaken based upon the recognition that, with good training and supervision, general nurses could provide surgery of the same quality as ophthalmologists [38]. The patients needing this intervention were primarily based in rural communities with little or no access to hospitals, so it seemed a sensible approach to provide services. However, studies assessing the productivity of these “trichiasis surgeons”, starting in 2007, showed that the median number of surgeries per surgeon per year was low—23 and 7, respectively, in Ethiopia [39] and Tanzania [40]. In Ethiopia, 41% of the trained trichiasis surgeons were no longer active after 2 years; of the active ones, the mean number of surgeries per surgeon per year was 41. These studies led to additional research in other settings which demonstrated similar findings [41]. Reasons for the exceptionally low level of productivity were traced to the lack of support for outreach, inadequate number of instruments and consumables, and inadequate supervision. Because of these findings, in most trachoma-endemic countries, there has been a shift back to the provision of trichiasis surgery by dedicated eye care personnel, generally ophthalmic nurses or ophthalmic clinical officers [42].

A final area which may be considered a task shifting strategy is the expectation that meaningful diagnosis and treatment of eye conditions can be provided at the primary health care level. Initially, primary eye care (PEC) concerned itself with preventable conditions, such as vitamin A deficiency and trachoma, but these are no longer important causes of vision loss in SSA. Twenty-five years ago, the usefulness of PEC in increasing the number of people presenting for cataract surgery and other sight-saving or sight-restoring interventions was questioned [26]. More recently, research has highlighted the inadequate skills of general primary care workers in recognizing and managing important eye conditions [43–45]. Considering the complexity and variety of conditions that can lead to vision loss, the paucity of tested curricula and treatment algorithms for this level, and the low numbers of patients with eye problems compared to other conditions demanding time from primary health care workers, it is unrealistic to expect much eye care to be delivered at the primary care level [46]. Instead, eye services at this level could more usefully focus on eye health education messages, health promotion, identification of abnormal eyes (without necessarily reaching a diagnosis), and clear referral guidelines. The provision of presbyopic spectacles to general health care services may be possible; a report from Zanzibar indicated that there was considerable support for this activity by health workers working in health centres although the actual number of spectacles dispensed was only 4.8 per health centre per month [47].

Overall, the existing research suggests that task shifting to generalists to provide eye care services needs to be approached cautiously. While there is likely a role of NPCS to support ophthalmologists in the provision of comprehensive eye care, an example of task sharing, the current expectations that “countries seeking to make rapid progress to improve CSR (cataract surgical rate) should prioritize investment in training new cataract surgeons over ophthalmologists…” is not justified by the evidence [37]. As far as the generalist trichiasis surgeons, recommendations following a scientific meeting in 2012 [42] have resulted in many programmes shifting back to using dedicated eye care staff to provide trichiasis surgery. Finally, there is little evidence to suggest that primary eye care following current training curricula that focus on diagnosis and treatment is a meaningful model within the general health care system in SSA [48, 49]. If any best practice examples exist or are developed, it is imperative that they be documented.

Clearly, there is little evidence to demonstrate that task shifting has been an effective solution to the perceived shortage of HReH in SSA. There is much that needs to be studied and understood about productivity of workers and the capacity of various cadres to deliver high-quality eye care that meets the demands of the population; this may be more sensible than rolling out more training initiatives.

## Conclusion

This paper has characterized the types of evidence that are needed to inform HReH target setting and policy in SSA and described the limited evidence that is available.

The variable epidemiology of eye conditions throughout SSA, the variety and complexity of diagnosis and management of these conditions, the evolving visual needs of populations, and differences in health systems among countries limit our ability to make sweeping, Africa-wide HReH policies and target setting. Promotion of one-size-fits-all HReH policies or targets for the 48 countries of SSA makes little sense. Instead, using the available information, individual countries should undertake a series of critical assessments. These should start with an assessment of current cadres providing eye care and the specific tasks they are trained to do related to each eye condition. The number of each cadre and their productivity in terms of services provided should be assessed and then measured against reasonable standards. Productivity may be limited by health system factors such as ineffective referral and by factors within HReH. Many eye surgeons are underperforming because the system does not provide them with enough patients. Reasons for this need to be investigated. If some surgeons are only doing surgery on blind cataract, further investigation as to whether this is due to their training or due to lower level staff acting as gatekeepers is needed. Investment to address productivity of existing surgeons would likely improve services without the additional cost of training new personnel. Training more people who are unproductive is a poor investment of resources. Investigating and understanding the reasons for low productivity must be a high priority. Additional research is needed to address the wide range of issues encapsulated within the variety of personnel included in HReH.

HReH policies adopted now regarding specific cadres should be viewed in the context of future anticipated eye care needs. Given the changing dynamics of both disease (increasing prevalence of diabetes and the need to address glaucoma) and patient need (desire for cataract surgery before blindness) and the complexity of eye care service delivery, promoting the widespread adoption of NPCS, whose training often equips them to operate only on late-stage cataract and falls short of equipping them to provide modern treatment for other surgical diseases (including glaucoma and diabetic retinopathy), does not make sense in modernizing SSA. On the other hand, in well-run systems headed by ophthalmologists where there are many patients, NPCS could free ophthalmologists to deal with more complex cases.

Above all, it is important to recognize that we are still learning about what works and what does not. A certain amount of flexibility is required to deal with this fact as well as the fact that medical technology and patient needs are evolving and will continue to evolve. SSA does not need nor deserve substandard eye care.

## Abbreviations

GAP: Global Action Plan
HReH: human resources for eye health
IAPB: International Agency for the Prevention of Blindness
MoH: ministry of health
NPCS: non-physician cataract surgeons
SSA: sub-Saharan Africa
WHO: World Health Organization
